# Capability for Fine Tuning of the Refractive Index Sensing Properties of Long-Period Gratings by Atomic Layer Deposited Al_2_O_3_ Overlays

**DOI:** 10.3390/s131216372

**Published:** 2013-11-28

**Authors:** Mateusz Śmietana, Marcin Myśliwiec, Predrag Mikulic, Bartłomiej S. Witkowski, Wojtek J. Bock

**Affiliations:** 1 Institute of Microelectronics and Optoelectronics, Warsaw University of Technology, Koszykowa 75, Warsaw 00-662, Poland; E-Mail: M.Mysliwiec@stud.elka.pw.edu.pl; 2 Centre de Recherche en Photonique, Université du Québec en Outaouais, 101 rue Saint-Jean-Bosco, Gatineau, QC J8X 3X7, Canada; E-Mails: predrag.mikulic@uqo.ca (P.M.); wojtek.bock@uqo.ca (W.J.B.); 3 Institute of Physics, Polish Academy of Sciences, Al. Lotników 32/46, Warsaw 02-666, Poland; E-Mail: bwitkow@ifpan.edu.pl

**Keywords:** optical fiber sensors, refractive index sensor, long-period gratings (LPG), Atomic Layer Deposition (ALD), thin films, optical properties, aluminum oxide (Al_2_O_3_)

## Abstract

This work presents an application of thin aluminum oxide (Al_2_O_3_) films obtained using atomic layer deposition (ALD) for fine tuning the spectral response and refractive-index (RI) sensitivity of long-period gratings (LPGs) induced in optical fibers. The technique allows for an efficient and well controlled deposition at monolayer level (resolution ∼ 0.12 nm) of excellent quality nano-films as required for optical sensors. The effect of Al_2_O_3_ deposition on the spectral properties of the LPGs is demonstrated experimentally and numerically. We correlated both the increase in Al_2_O_3_ thickness and changes in optical properties of the film with the shift of the LPG resonance wavelength and proved that similar films are deposited on fibers and oxidized silicon reference samples in the same process run. Since the thin overlay effectively changes the distribution of the cladding modes and thus also tunes the device's RI sensitivity, the tuning can be simply realized by varying number of cycles, which is proportional to thickness of the high-refractive-index (*n* > 1.6 in infrared spectral range) Al_2_O_3_ film. The advantage of this approach is the precision in determining the film properties resulting in RI sensitivity of the LPGs. To the best of our knowledge, this is the first time that an ultra-precise method for overlay deposition has been applied on LPGs for RI tuning purposes and the results have been compared with numerical simulations based on LP mode approximation.

## 1. Introduction

Long-period gratings (LPGs) have been known now for almost two decades [1]. LPGs are a periodic modulation of the refractive index along the length of the optical fiber. Under special phase-matching conditions, the grating couples the fundamental core mode to discrete cladding modes that are rapidly attenuated due to absorption and scattering. The coupling from the guided mode to cladding modes is wavelength-dependent, so one can obtain a spectrally selective loss. For the transmission spectrum of the LPG structure, two parameters can vary under the influence of an external stimulant: the resonance wavelength and the resonance transmission. The sensitivity is then typically defined as a shift of the resonance wavelength induced by a measurand [2]. A shift of resonance has been reported under a number of external influences including temperature, pressure, strain, bending, and refractive index (RI) (see e.g., [2,3]).

Due to coupling of the cladding modes by the LPG structure, there is a significant dependence between the properties of the external media and the spectral response of the LPG. The highest sensitivity of the LPG to the external RI can be observed when the external medium's RI value is close to that of the cladding, which is typically made of fused silica (*n*_D_ = 1.458). However, it has been shown that high-refractive-index (high-*n*) thin overlays can tune the intrinsic sensitivity of the LPG devices to a certain range of external RIs (*n_ext_*) [4]. A specially designed coating, in terms of its thickness and optical properties, optimizes the interactions of the light guided in the fiber and in the coating [4–7]. By modifying the guiding conditions of the cladding modes in this way, we can make the LPG highly sensitive in a narrow but arbitrary range of *n_ext_*, while making it insensitive to the *n_ext_* when other parameters are measured [8]. In order to fulfill durability requirements for the LPG-based sensor, the coating cannot change its properties under either long-term presence in the liquid or other harsh environmental conditions, including temperature variations and mechanical surface cleaning.

To date successful tuning of the RI response through the use of nano-coatings has been reported by a number of authors, who mainly employed various liquid-precursor-based deposition techniques, such as Langmuir-Blodgett (LB) [4], sol-gel [5], and electrostatic self-assembly (ESA) [9] also called as ionic self-assembled multilayers (ISAM) [10]. In our previous works we reported results on modification of the RI response of the LPG-based sensors with hard and high-*n* overlays deposited from gas precursors with a radio-frequency plasma-enhanced chemical-vapour-deposition (RF PECVD) method [6,8]. The method allows for efficient deposition of thin films with well defined properties. The properties of the films can be easily changed over a wide range of *n* values by varying the gas composition and other deposition parameters [11]. However, the control of the film thickness deposited with the method in nanometer range, as well as symmetrical deposition around the fibre with RF PECVD method are still challenging.

Atomic Layer Deposition (ALD) is currently assumed as one of the most promising thin film deposition techniques for the fabrication of nano-devices [12]. The ALD method was developed for making thin film electroluminescent displays, and since then it has become a part of the standard processing technology in the semiconductor industry. The method is based on the gas-solid reactions occurring at the surface of a substrate, and belongs to a group of layer-by-layer techniques. Its distinctive features among CVD methods are self-limitation of precursor adsorption and its pulsed character. The deposition procedure of a single bilayer consists of the following steps: substrate exposure to gas precursor, pumping out of the precursor and other products from the chamber, exposure to reactant species, and second pumping out of the chamber. The surface reactions can be initiated thermally or supported by plasma [13]. A wide range of materials including metals and dielectrics can be deposited for different purposes [14]. The process temperature is typically in range of 30 to 150 °C, depending on the precursor [14]. Besides the growth rate, that is well controlled, the deposited films are extremely conformal over the surfaces. The technique is usually considered as feasible only for very thin films, but the low growth rate can be compensated with the easily increased size of the reactor and multiplication of the substrates. The technique offers similar advantages to the ESA/ISAM techniques. Due to better possibility of penetration of extremely small trenches by gas than by liquids, the method can be used, e.g., for coating internal surfaces of photonic crystals. The method is still rarely applied to optical fibres [15–17].

In this paper we present a successful deposition of aluminium oxide (Al_2_O_3_) thin overlays on LPGs using the ALD technique. In our previous work we applied zinc oxide (ZnO) thin films, the but robustness of the films in aquarius solution is limited [17]. The Al_2_O_3_ shows high hardness, as well as high temperature and electrical resistance. The material has been applied in mechanically and chemically protective coatings, gas diffusion barriers, heat-resistant components and electric insulators. In this work the Al_2_O_3_ films are applied for fine tuning the spectral properties of the LPGs, including their RI sensitivity over its wide range. The Al_2_O_3_-containing films deposited with the ESA technique have already been applied for the formation of nano-resonators on the fibre end-face [18], as well as tuning of LPGs [19]. The applied film was a composition of cationic polyelectrolyte containing Al_2_O_3_ and a polymer playing the role of anionic polyelectrolyte. It must be emphasized that the ESA/ISAM layer formation mechanism where polyelectrolytes in liquid form are required, limits resolution of the control of thickness of the film to a couple of nm [9,10,20]. Monolayer thickness of 2.6 nm has been reported when using the LB technique [4]. Moreover, since the film material is a compound containing Al_2_O_3_, its properties may significantly differ from those of Al_2_O_3_. The Al_2_O_3_ applied in this work, can be tuned in sub-nm scale thanks to advantages of the ALD method. For novel LPG-based sensors, especially those working at the dispersion turning point, high precision in determination of overlay properties is highly desirable [10,21–23].

For simulation of the LPG response to overlay deposition and to variation of *n_ext_*, we used a linearly polarized (LP) mode approximation, which is assumed to be valid for low *n* contrast between the fibers and overlay materials [20,24]. The obtained results have been compared with experimental data.

## 2. Experimental Details

### 2.1. LPG Fabrication

In this experiment we used commercially available Corning SMF28 optical fiber (Corning Incorporated, Corning, NY, USA). A set of LPGs was written with a computer-assisted precision arc-discharge apparatus described in [3]. The discharge current was adjusted to be low enough to heat the fiber locally and not to produce any visible tapers due to the axial tension applied to the fiber. The grating period was set to Λ = 500 µm. The optical transmission of the fiber in the range of λ = 1.160 – 1.660 nm was monitored during the LPG fabrication process in order to obtain the desired spectral attenuation notches. We used an Agilent 83437A broadband light source and an Agilent 86142B optical spectrum analyzer (Agilent Technologies, Inc., Santa Clara, CA, USA) for this purpose.

### 2.2. Al_2_O_3_ Nano-Film Deposition and Characterization

The Al_2_O_3_ thin films were deposited on the LPGs and reference oxidized silicon wafers using the Savannah-100 ALD system (Cambridge Nanotech, Waltham, MA, USA). The LPG samples were cleaned in isopropanol, while the Si wafers went through the Radio Corporation of America cleaning procedure [25]. Then the Si wafers were thermally oxidized in order to receive SiO_2_ on their surface, which is assumed to be a good reference for fused silica fibers. For the Al_2_O_3_ deposition processes, water and trimethylaluminum (Al(CH_3_)_3_, TMA) were used as an oxygen and aluminum precursors, respectively. Between gas pulses the chamber was purged with nitrogen. Thickness of the Al_2_O_3_ films in the range of 100 up to 250 nm was changed by controlling the number of cycles of the ALD process (900 to 1,800). The temperature during the processes was set to 150 °C.

The properties of the Al_2_O_3_ films including their thickness (*d*), refractive index (*n*), and extinction coefficient (*k*) were measured on reference wafers by means of Horiba Jobin-Yvon UVISEL spectroscopic ellipsometer *SE* (Horiba Scientific, Edison New Jersey, NJ, USA) in the range λ = 260 to 2,060 nm. To fit the measurement data to a physical model, a three-layer model (Si wafer/SiO_2_/Al_2_O_3_) was used where a single-layer Forouhi-Bloomer dispersion formula [26] of Al_2_O_3_ film was applied and fitted with mean-square error χ^2^ < 3. The thickness of the SiO_2_ layer was measured before the Al_2_O_3_ deposition to be 99.7 nm.

Moreover, the thicknesses of the deposited films have been directly controlled on the fibers by cleaving some of the samples and by measuring the thickness at the fiber cross-section using a Hitachi SU-70 Scanning Electron Microscope *SEM* (Hitachi High-Tech, Tokyo, Japan).

### 2.3. LPG Sensing Experiment

The RI response of the LPGs has been measured for samples immersed in glycerin/water mixtures with n_D_ from 1.3330 to 1.4722 RIU. The n_D_ of the liquids was determined using a VEE GEE PDX-95 refractometer (VEE GEE Scientific, Inc., Kirkland, WA, USA) working with an accuracy of ±10^−4^ RIU. The LPGs were kept under the same tension and temperature during all the experiments.

### 2.4. Numerical Simulations of LPG

The optical simulations were performed using the Optigrating v.4.2 software (Optiwave Systems, Inc., Ottawa, ON, Canada). The software employs LP mode approximation and coupled-mode theory [7,24]. The model of the grating and the fiber is based on fiber datasheet, and grating period is determined during the fabrication process. The model has been adjusted up to experimental data by changes of core and cladding diameter [27]. We assumed dispersion curves of *n* for each of the Al_2_O_3_ films and water according to *SE* analysis and data given by Damion *et al.* [28], respectively.

## 3. Results and Discussion

### 3.1. Properties of the Al_2_O_3_ Nano-Overlay

**Figure 1.**
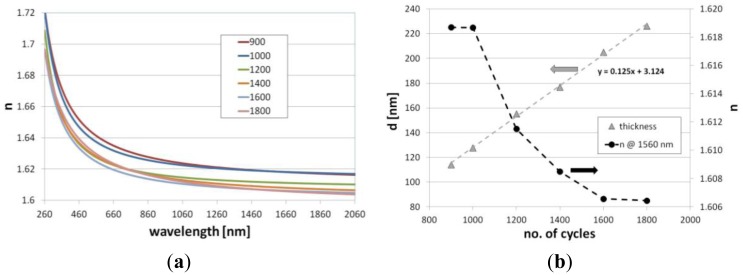
Properties of the Al_2_O_3_ films deposited on the oxidized Si reference wafers for 900 to 1,800 deposition cycles where (**a**) shows dispersion of refractive index *n* and (**b**) thickness of the films *d* and their *n* at λ = 1,560 nm.

Spectral response of the LPGs to *n_ext_* highly depends on optical properties and thickness of the overlays [7]. The variation in properties of the Al_2_O_3_ films deposited on reference Si samples with the number of deposition cycles is shown in Figure 1.

The results prove that thickness of the films deposited with the ALD method can be determined with a sub-nm precision. According to the measurements, thickness of the film increases linearly with the number of cycles and reaches a deposition rate of 0.125 nm/cycle. Moreover, a slight variation in the optical properties of the films with the number of the cycles can be observed. Since the extinction coefficient *k* of the films is close to 0, we show here only dispersion of *n* (Figure 1a). In the infrared spectral range (λ = 1,560 nm) the *n* decreases with the number of cycles by over 0.012 RIU, especially in the range between 1,000 and 1,600 cycles (Figure 1b). The effect can be related to a modification of internal structure of the film or to a stress in the films induced by the substrate with the increase of their thickness [29]. The phenomenon remains in contradiction with the variations of *n* for the films deposited with other CVD methods where the increase of the film thickness is typically followed by an increase of *n* [30]. Since in other vapor-based thin film deposition methods plasma or high temperature modifies the film during the deposition, in case of the ALD more likely the substrate effect induces a decrease of the *n* with the film thickness. Both variations in thickness and optical properties of the overlay are critical from the point of view of the LPG-based structure performance and they must be taken into account in the sensing device design process [10].

**Figure 2.**
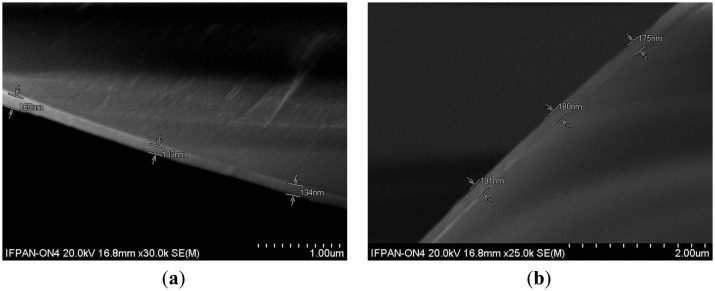
Cross-section of the optical fibers with deposited Al_2_O_3_ overlays, where thicknesses for (**a**) and (**b**) films on reference Si samples are 127 and 177 nm, respectively.

Thickness of the films on reference samples has been compared to the thickness of the overlays obtained on optical fibers when their cross-section was investigated (Figure 2). It can be seen that the film thickness on both cylindrical optical fibers and on flat reference samples is very similar. Since the precision of thickness estimation with the *SEM* is limited, we can assume a very good agreement between the measurement of films properties on optical fibers and on the reference samples.

### 3.2. Simulations of an Effect of the Nano-Overlay Deposition

**Figure 3.**
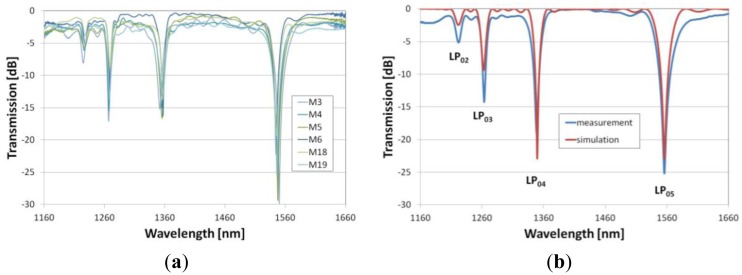
Spectral response of the LPGs measured before deposition of the overlays where (**a**) shows response of selected set of LPGs (samples M3 to M19) and in (**b**) measured and simulated spectral response of a representative LPG before deposition of the overlays are compared. Resonances coming from coupling of the cladding modes from LP_02_ to LP_05_ are marked.

For deposition of the films on the LPGs, a set of gratings has been selected showing a very similar spectral response (Figure 3a). The gratings have resonances at about 1,200, 1,260, 1,350 and 1,550 nm, coming from coupling of LP_02_, LP_03_, LP_04_, and LP_05_ cladding modes, respectively. The slightly different spectra of the gratings are caused by a limited repeatability of the fabrication procedure. It has been shown that even a very small tapering of the fiber during the arc-induced fabrication process can have a significant influence on the spectrum of the LPGs [27]. Despite the small differences between the gratings fabricated with the arc-method, LPGs are highly resistant to high-temperature post-processing and that is why they are assumed to be a good platform for the experiment [31]. Based on the measured spectra a numerical model of the LPG has been developed. The fitting procedure involved a slight adjustment of the core's *n* and diameters of both core and cladding. As a result of the fitting, a very good agreement between measured and simulated spectra has been obtained (Figure 3b).

**Figure 4.**
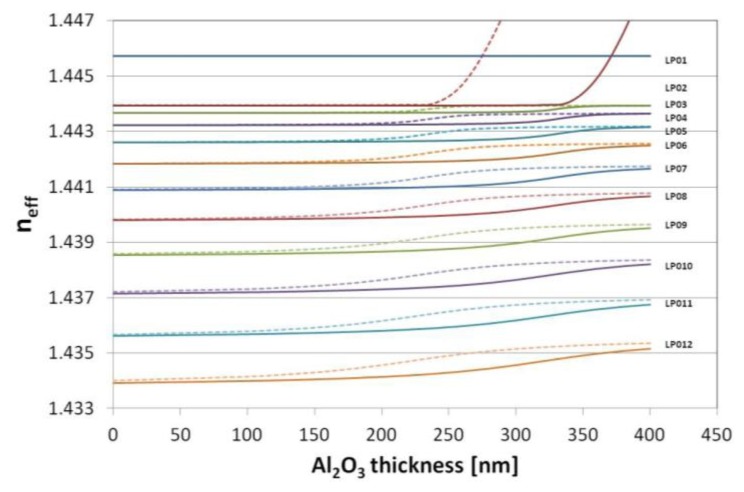
Simulated variation of the effective refractive index at λ = 1,550 nm of the modes LP_01_ to LP_012_ when *n_ext_* was 1 (solid lines) and 1.318 (dotted).

**Figure 5.**
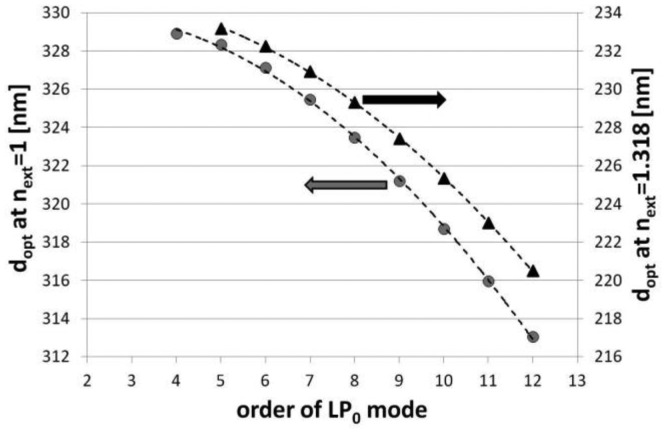
Calculated *d_opt_* of the Al_2_O_3_ overlay where mode transition takes place for each of the cladding modes and the LPG is surrounded by air (*n_ext_* = 1 RIU) and water (*n_ext_* = 1.318 RIU).

Based on the numerically modeled influence of the Al_2_O_3_, an overlay thickness on the effective refractive indices (*n_eff_*) of the modes up to LP_012_ has been calculated at λ = 1,550 nm. We assumed here the value of *n* of the overlay as for 1,200 cycles-long deposition (*n* = 1.611 RIU, shown in Figure 1). When *n_ext_* is set to 1 (as for air) and 1.318 RIU (as for water at λ = 1,550 [28]), the transition of the modes, resulting in propagation of the lowest order cladding mode (LP_02_) in the overlay, takes place above 330 and 230 nm of the Al_2_O_3_ overlay thickness, respectively (Figure 4). The phenomenon induces a reorganization of all the cladding modes and for higher order modes the transition occurs for the lower overlay thicknesses. Since at the mode transition conditions, the LPGs show the highest sensitivity to variations in the *n_ext_*, the properties of the films must be precisely adjusted for certain range of the *n_ext_*. When the transition is assumed to take place at *n_eff_* reaching half between of each mode when no coating is applied [7], the approximated optimum thickness (*d_opt_*) of the Al_2_O_3_ overlay for each mode is plotted in Figure 5. Significantly thinner overlays are required when coupling of higher order cladding modes is taken into consideration for sensing purposes. The determination of properties of the overlays with a high precision as offered by the ALD technique is than highly requested.

### 3.3. Refractive Index Sensitivity

**Figure 6.**
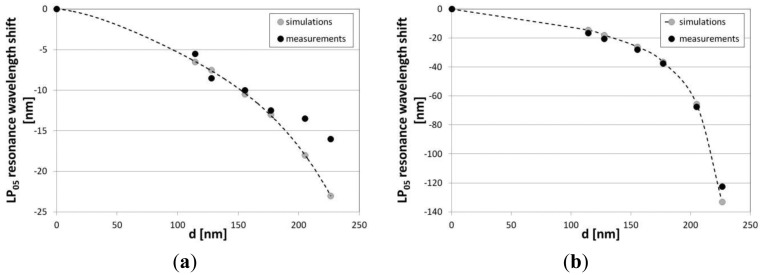
Measured and simulated LP_05_ mode resonance wavelength shift with thickness *d* of the Al_2_O_3_ overlay when LPG is surrounded by (**a**) air (*n_ext_* = 1) and (**b**) water (*n_ext_* = 1.318 RIU at λ = 589 nm and *n_ext_* = 1.3330 RIU at λ = 1.550 nm for simulations and experimental data, respectively).

Results of the measurements and simulations have been compared next. A good agreement between theoretical and experimental data for the resonance LP_05_, which shows the highest sensitivity, can be seen in Figure 6. In our measurements and simulations we had to take into account the dispersion of *n_ext_*. The value of *n_ext_* in visible range (λ = 598 nm) significantly differs from the one in infrared (λ = 1,550 nm). In case of water, the difference reaches almost 0.015 RIU and cannot be neglected when precise measurements are discussed and compared with the results of simulations. When thickness of the Al_2_O_3_ overlay increases, the resonances experience a shift towards lower wavelength. An increase in the overlay thickness induces an increase in the effective refractive index of *m*th cladding modes ( $n_{\text{eff}}^{0m}$), and according to the fundamental Equation (1) for the LPGs, where Λ is the period of the grating and $n_{\text{eff}}^{01}$ is effective refractive index of the propagating core mode, causes a decrease in the resonance wavelength ( $\lambda_{\text{res}}^{m}$):

(1) $$\lambda_{\text{res}}^{m}=\Lambda \left(n_{\text{eff}}^{01}-n_{\text{eff}}^{0m}\right)$$

**Figure 7.**
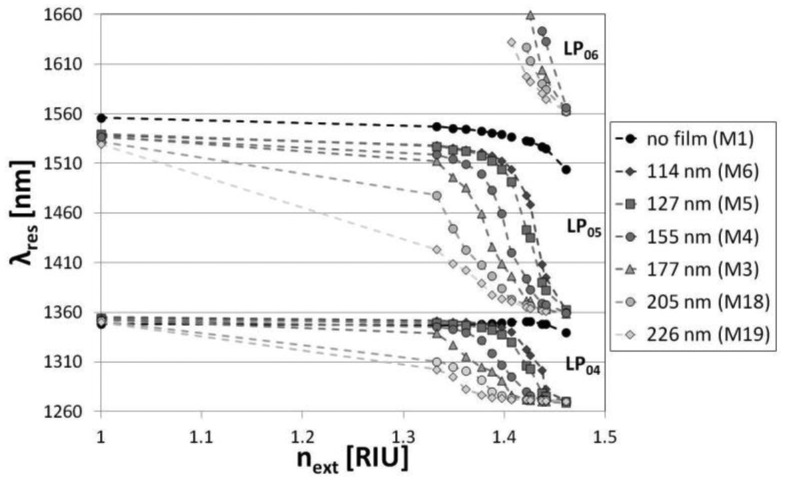
Resonance wavelength shift with *n_ext_* for the samples with Al_2_O_3_ film of different thickness. The *n_ext_* is given at λ = 589 nm.

**Figure 8.**
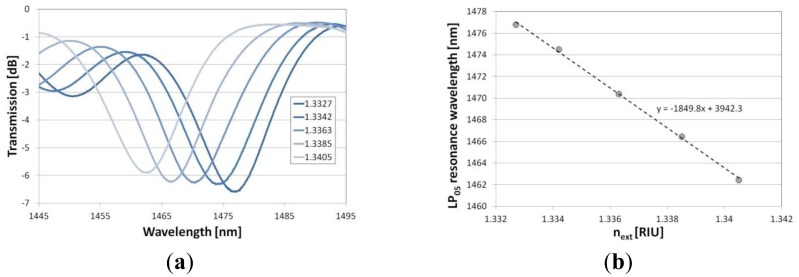
Response of the LPG (sample M18) with Al_2_O_3_ overlay (205 nm in thickness) to variations in *n_ext_* in the range close to that of water where (**a**) shows shift of the spectrum and (**b**) shift of the resonance wavelength for each spectrum. The *n_ext_* is given at λ = 589 nm.

A slight disagreement between the measurements and simulations can be seen for the thicker overlays, *i.e.*, 205 and 226 nm in thickness. The phenomenon may be related to the decrease in *n* of the Al_2_O_3_ film with its thickness. When the conditions are close to the mode transition, even a slight disagreement of overlay thickness and *n* matters. Also a slight disagreement between the simulated and measured LPG spectrum before the deposition may have an effect on the discrepancy. The response of the LPGs to the variations in *n_ext_* has been investigated next. It can be seen in Figure 7 that the Al_2_O_3_ overlay greatly modifies the response depending on properties of the overlay. Increasing the thickness or *n* of the coating can shift the maximum RI sensitivity to its lower values, while decreasing the sensitivity to the higher *n_ext_* [8]. We successfully shifted the maximum sensitivity of the LPGs from an *n_ext_* of 1.458 RIU (for a grating with no film) to one in the range between 1 and 1.458 RIU. For samples M19 surrounded by water, the coating is thick enough or has high enough *n* to induce the mode transition in the *n_ext_* range below the one of water. The range of the highest RI sensitivity can be easily adjusted by a number of overlay deposition cycles. When the overlay thickness reaches 205 nm, the highest sensitivity for the LP_05_ mode is in the *n_ext_* range close to that of water (Figure 8). The sensitivity in this range is highly requested when label-free bio-sensing applications of the LPGs are considered [32]. For the M18 grating with the overlay thickness of 205 nm, the RI sensitivity, defined as the LP_05_ mode resonance wavelength shift with *n_ext_*, reaches −1,850 nm/RIU, which is over 20 times higher than for the comparable LPG with no coating (−90 nm/RIU) in the same *n_ext_* range, *i.e.*, 1.3330 to 1.342. Since the RI sensitivity is known to increase with the order of the cladding modes [2], and the ALD method allows for relatively low temperature deposition [14], the sensitivity can be further improved by using the gratings induced with a lower period using a UV-writing method [31].

## 4. Conclusions

In this paper we introduce atomic layer deposited Al_2_O_3_ overlays onto LPG-based sensing structures. We show that by a proper selection of the deposition process cycles one can successfully tune the spectral responses of the LPG sensors and their RI sensitivity range. The method allows for precise determination of the thickness of the overlays on a sub-nm scale. We show that the refractive index of the films decreases slightly with the number of the deposition cycles, and the effect can have an influence on the LPG-based device functional properties. Taking into consideration the excellent optical, mechanical and chemical properties of Al_2_O_3_, namely its low optical absorption, high wear resistance and chemical inertness, the coating can clearly be acknowledged as a good overlay material for the robust LPG-based sensing devices, including biosensors working at dispersion turning point. Moreover, simulations of the LPGs with the overlays performed using the LP mode approximation stay in good agreement with the experimental data, especially for the thinner films (<200 nm).

Due to the fact that the high-*n* coatings can be deposited thinner and still have a comparable effect to the thicker coatings with a lower *n*, other materials able to be deposited with the ALD method can be also considered as overlays for the LPGs. Deposition of, e.g., HfO_2_ (*n_D_* = 2), ZrO_2_ (*n_D_* = 2.1) or TiO_2_ (*n_D_* = 2.4), will be less time-consuming and will result in the similar effect to the Al_2_O_3_ deposition, and will simultaneously allow for sub-nm precision in determination of the overlay thickness thanks to the advantages of the ALD technology.
